# Editorial: Matricellular Receptors As Potential Targets in Anti-Cancer Therapeutic Strategies

**DOI:** 10.3389/fphar.2016.00095

**Published:** 2016-04-14

**Authors:** Hervé Emonard, Laurent Duca, Stéphane Dedieu

**Affiliations:** ^1^Centre National de la Recherche Scientifique UMR 7369, Matrice Extracellulaire et Dynamique CellulaireReims, France; ^2^Laboratoire de Signalisation et Récepteurs Matriciels, Université de Reims Champagne-Ardenne, UFR de Sciences Exactes et NaturellesReims, France

**Keywords:** discoïdin domain receptor (DDR), elastin receptor, integrin, low-density lipoprotein receptor-related protein (LRP), syndecan, thrombospondin receptor, tyrosine kinase receptor, urokinase receptor

Throughout their life, tumor cells proliferate, migrate, overcome obstacles and survive. All these actions require multiple interactions between tumor cells and their extracellular surroundings through specialized cell-surface molecules termed matricellular receptors. We define the matricellular receptors as being receptors that bind extracellular matrix (ECM) structural proteins or soluble factors that dynamically act on ECM homeostasis. Matricellular receptors mediate signalings from the extracellular environment to cell nucleus and drive main biological functions that are cell growth, survival and migration. Numerous data from the last decade provide evidence that matricellular receptors are biosensors that allow to a tumor cell to answer to microenvironmental variations. In this sense they are important contributors to tumor cell malignancy.

Tumor development is associated with an intense remodeling of ECM that generates biologically active fragments, termed matricryptins. Ricard-Blum and Vallet review on matricryptins and their receptor(s) and co-receptor(s), which form a complex network at the surface of tumor and stromal cells. They describe their roles in angiogenesis, tumor growth and metastasis, and their anti-cancer drug potential.

Interaction between cells and the ECM largely involves the well-known cell-surface receptors integrins. Data accumulation during the last 20 years demonstrated that these heterodimeric proteins act as sensors of cell microenvironment by transducing intracellular signals regulating cell fate. They are now considered as critical players in cancer progression. Blandin et al. summarize the current knowledge about integrin involvement in tumor progression and specifically provide informations about β1 integrins as therapeutic targets to disrupt hallmarks of cancer.

Beside integrins that bind various ECM macromolecules, the tyrosine kinase receptors Discoidin Domain Receptors (DDR) specifically interact with collagens. Collagens, mainly the fibrillar type I collagen, are major components of the tumor stroma. After summarizing biochemical data on DDR, Rammal et al. pinpoint the roles of DDR1 and DDR2 in the successive phases of a cancer development. Finally, the authors review pharmacological approaches to inhibit DDR1 and DDR2, which might represent valuable targets for anti-cancer therapies.

Elastin is the longest-lived protein in vertebrate and provides elasticity to tissues with high mechanical constraints such as lung or skin. Its degradation during cancer progression not only affects its mechanical properties but also generates elastin-derived peptides (EDP) that are actively involved in the development of cancer. Scandolera et al. describe the role of EDP in tumor development and focus their review on the main elastin receptor, an heterotrimer named the Elastin Receptor Complex (ERC), unique by its composition and operating mechanism. They propose anti-ERC therapeutic strategies and describe ERC involvement in cancer-associated processes such as diabetes and thrombosis.

Syndecans are transmembrane proteoglycans expressed at the cell-surface of various cell types. Syndecans are now considered as key regulators of tumorigenesis and cancer progression, especially as being involved in the control of cell proliferation, migration and angiogenesis and in cell-matrix interaction and dynamics. In this regard, the review by Cheng et al. discusses the current state of knowledge of syndecans expression and implication in the field of cancer, with a special and exciting focus on syndecans binding with PDZ domain-containing proteins. The regulation of PDZ binding by phosphorylation of the syndecan cytoplasmic tail is notably debated. Consistently, the experimental data reported by Kashyap et al. focus on syntenin, a scaffold protein containing two PDZ domains and known as an intracellular adaptor for syndecans. To evaluate the potential benefit of anti-syntenin strategies, the authors report the effects of syntenin depletion on various cancer cells from distinct origins. Their results show that syntenin loss of function leads to a significant decrease in tumor cell proliferation, growth and migration in each cancer cell model with a noteworthy defect in the cell-surface expression of active β1-integrin. The authors conclude that syntenin may constitute a molecular target of pharmacological interest in the tumor context.

Growth factors receptors are recognized as critical players in tumor progression by regulating diverse biological activities such as proliferation, migration or survival through their binding on Tyrosine Kinase Receptors (TKR). Among this large family, Erb receptors are often overexpressed, amplified, or mutated in many forms of cancer, making them important therapeutic targets. In their review, Appert-Collin et al. describe the regulation of Erb activity and their role in epithelial-mesenchymal transition. They illustrate the dedicated therapeutic strategies allowing their inhibition with an interesting focus on peptides which mimick transmembrane domains. Beside their role in cancer progression, TKR are involved in the development of various fibrotic diseases. In this regard, these diseases also benefit from advances in the pharmacological strategies developed to fight cancer. Particularly, VEGFR, PDGFR, FGFR, and EGFR kinases appear as potential targets for anti-liver fibrosis therapies. Qu et al. summarize the anti-liver fibrosis effects of multitargeted TK inhibitors and molecular mechanisms. with a specific focus on anti-cancer drugs such as sorafenib and erlotinib.

The urokinase receptor (uPAR) is a key cell-surface receptor generating pericellular proteolysis involved in tissue remodeling processes and triggering intracellular signaling pathways to support various cancer-related events. The mini review by Gonias and Hu gives a synthetic overview of uPAR expression and function in cancer and provides a relevant schematic representation of uPAR-related mechanisms at both sides of the cell membrane. The authors also address the role of uPAR in the tumor cell resistance to anti-cancer drugs. This part is quite new and attractive and constitutes an exciting area to explore for the future.

While numerous matricellular receptors only exhibit signaling properties, the low-density lipoprotein receptor-related protein-1 (LRP-1) further has endocytic capacities. Van Gool et al. provide an overview of this complex receptor, with a particular focus on the multiple roles played by LRP-1 in cancer progression. Furthermore, the authors present recent (pre)clinical data that suggest applications of LRP-1 as therapeutic tool (in brain cancers, for crossing the blood-brain barrier) and diagnostic/prognostic tool. The amount of LRP-1 at the cell surface is strictly regulated by a proteolytic process termed shedding, which is itself controlled, notably by cell cholesterol level. Dekky et al. present original data that highlight the importance of cell cholesterol distribution in the modulation of LRP-1 shedding. Their results suggest an inverse correlation between intracellular cholesterol concentration and LRP-1 shedding efficiency.

As we have mentioned, these matricellular receptors may constitute relevant targets to fight against malignant diseases. As examples, Jeanne et al. focus on the CD47 and CD36 molecules that function as cell-surface receptors for thrombospondin-1, a large matricellular glycoprotein highly overexpressed within tumor stroma where it promotes an aggressive phenotype. The authors review the various therapeutic options, including antibody-based approaches, therapeutic gene modulation and TSP-1-derived peptides and mimetics. Interestingly, the authors also discuss in detail the more recent and innovative approaches including combination strategies to improve radiotherapy and chemotherapy.

Through (mini) reviews and original reports, this Research Topic highlights matricellular receptors that could represent valuable targets for the development of original anti-cancer strategies. We would like to thank all the authors for their important contribution in these exciting fundamental and applied research fields. We are also grateful to the reviewers and editors for their constructive comments that allowed our issue to reach a high standard quality.

## Author contributions

All authors listed, have made substantial, direct and intellectual contribution to the work, and approved it for publication.

### Conflict of interest statement

The authors declare that the research was conducted in the absence of any commercial or financial relationships that could be construed as a potential conflict of interest.

